# Selections and Abstracts

**Published:** 1903-12

**Authors:** 


### *SELECTIONS AND ABSTRACTS.*

---

### Other Sources of Typhoid Infection Than Through the Medium of Drinking Water, and How to Guard Against Them.*

I am very glad, indeed, that our president, in assigning my theme as one of the "regular reporters" for this meeting of the State Medical Association, selected that of "Other Sources of Typhoid Infection Than Through the Medium of Drinking Water, and How to Guard Against Them," because from my observation, and from the recent researches made in large epidemics of typhoid fever, I am convinced that the medical profession greatly exaggerates the part that drinking water plays in the dissemination of that disease. The subject is, therefore, a "congenial" one to me.

Sanitarians, in writing learned theses on the etiology of typhoid, are wont to make such statements as the following: "Show me a city's mortality statistics from typhoid, and I will tell you the character of its water supply," and "from any given case of typhoid fever the cause can, and should be, discovered"; but the fact remains that in very many epidemics, and in more of sporadic cases, the cause is frequently from other source than drinking water, or it is not discovered at all. Recent investigations seem to show that, except in the cities, the germs of typhoid are disseminated more in other ways than through the medium of drinking water. I do not mean to deny that typhoid fever is largely a water-borne disease, but in this article I am not expected to discuss its spread through drinking water—all of us know too much about that side of the question—though in considering the other sources of typhoid infection, I shall mention some methods of conveying the disease where polluted water is indirectly the cause, though the infection is not taken into the system in drinking water.

The great epidemics of typhoid fever that visited the concentra-

---

*Read by Seale Harris, M.D., before the Medical Association of the State of Alabama, April, 1903. Reported in the Mobile Medical and Surgical Journal.

tion camps of the United States volunteer army during the Spanish-American war, in 1898, were carefully studied by a board of army surgeons, consisting of Majors Walter Reed, Victor C. Vaughan and Edward O. Shakespeare. In ninety-two regiments investigated there was a total number of 107,973 men; 20,738, or nearly 20 per cent., developed typhoid fever; and of these, there were 1,580 deaths—a mortality rate of 7 per cent. of those who contracted the disease. This board examined the records of all these cases, they visited all the cities where the camps were located, they investigated carefully the source and quality of the water supplies of all the camps, also their food supplies, and the methods of disposing of their excreta—in fact they investigated everything that could have any bearing upon the cause and spread of typhoid fever among our soldiers, more of whom suffered and died from typhoid fever than from Spanish bullets. All the members of this board were sanitarians of renown and great experience, and having been given every possible facility for making investigations, their conclusions are of great value and have almost revolutionized our ideas regarding the origin and spread of typhoid fever. Their report, which was published by the Government and which may be obtained free by application to the Surgeon-General of the army, is one of the most valuable contributions to the literature of typhoid fever that has been published in years. Their report presents so forcibly some of the facts which I desire to bring out that I shall quote freely from it. Among the general conclusions of this board are the following statements:

"Infected water was not an important factor in the spread of typhoid fever in the national encampments in 1898."

"It may be stated in a general way that the number of cases of typhoid fever in the different camps varied directly with the methods of disposing of the excretions."

According to these distinguished authorities on typhoid, the most active agents in the dissemination of the disease in over 20,000 cases were: Flies, personal contact, and dust, though they thought that in some of the camps the drinking water was infected.

There are many sources of typhoid infection other than through the medium of drinking water, the more important of which I shall discuss under separate headings.

*Milk.* Infected milk is a fruitful source of typhoid fever. Eberth's bacillus will thrive and multiply in milk without affecting its taste or appearance. It will live for days even in sour buttermilk, and fresh milk is an excellent culture medium for the germ. Some have thought that the germ can be ingested by the cow in drinking water and be excreted in the milk without destroying its vitality. However, this is hardly probable, unless the cow actually has typhoid fever, and there is no evidence to prove that cattle are subject to the disease. It seems possible that the germs might be taken into the stomach in water, and not having been destroyed in the alimentary canal of the cow, be excreted in the feces, but the germ has never been demonstrated in the excreta of the cow. Bacterial examination of milk has frequently shown the colon bacillus, and it seems possible that the typhoid germ could infect milk in the same way; that is by the udders becoming soiled with the feces containing the germ.

Milk may be infected in any of the following ways: From the udder or teats of the cow, from the hands of the milkman, from milk cans and pans which have been washed in infected water, from milk diluted with infected water by unscrupulous dairymen, from dust holding in it the typhoid bacillus, and by infected flies.

Pastures for cows are commonly located in the marshes and lowlands, where the soil is fertile and the grasses grow luxuriantly and the drainage from the surrounding country, or from the town, flows into the shallow streams which traverse them. When the rains come they wash the sewage into these streams, the pastures are flooded, and puddles of water remain for some time. The cows go into the streams for water, or lie down in the puddles, which, if infected, can readily in many ways infect the udder or teats and eventually infect the milk. If the milkman happens to be nursing a case of typhoid fever, and is not as careful as he should be in disinfecting his hands, he can easily infect the milk. A number of cases have been traced to this source. If the milk vessels are cleansed in polluted water, as can be done for instance by washing them in the shallow streams in the pasture, they can infect the milk contained in them.

During my college days an epidemic of typhoid fever occurred among the students who boarded at a certain hotel, fifteen or twenty of whom contracted the disease. They used the same drinking water that was drunk by more than five hundred other students. A committee from the faculty was appointed to inspect the hotel premises, but nothing was found there that could account for the epidemic. Upon further investigation it was found that the cows which belonged to the proprietor of the hotel was pastured in a lowland, through which traversed a shallow stream. The cows were milked in the pasture; their udders and milk vessels were cleansed in the water from this infected stream. There had been some cases of typhoid fever in houses located above and near the stream, and evidently the rains washed the excreta into the stream which flowed through this meadow, thus polluting the water and indirectly the milk. The committee reported that the epidemic was probably due to infected milk, whereupon the proprietor discontinued the use of the milk from the cows which were pastured in this meadow, and no further cases of typhoid developed among the students.

In the towns and villages, most of which have no sewerage systems, the cows are almost invariably pastured in the lowlands of the suburbs of the town, where all the filth and impurities drain directly into the streams that supply the cows with water. It certainly seems reasonable that the milk can become contaminated in some of the ways that I have mentioned, and I am convinced that much of the typhoid fever in such towns and villages, as well as in the rural districts, is due to infected milk.

*Flies.* The common house fly has been shown to be a frequent carrier of the typhoid germ. He has for many years been under suspicion of being guilty of that crime, but he has been recently found guilty beyond a reasonable doubt, and has therefore been condemned to death, or to banishment from homes which would be free from typhoid fever.

Majors Firth and Horrocks, two British army surgeons, examined flies, which had been allowed to crawl over typhoid stools mixed with fresh cultures of the bacilli, and all of those examined contained the germs of typhoid on their legs, heads, wings or bodies. It has been supposed that the fly may take the germs into his stomach and give them off in his excrement, but these observers failed to find the typhoid germs in any of the excreta of flies that had been fed on typhoid stools.

The observations of Dr. Alice Hamilton, of Chicago, are of more practical value, and they leave no doubt but that the fly is a carrier of the typhoid germ. During the recent epidemic of typhoid fever in Chicago, she examined the conditions in the locality where there were the greatest number of cases. She caught flies from two undrained privies, on the fences of two yards, on the walls of two houses, and in the room of a typhoid patient. These flies were placed in eighteen sterilized tubes containing suitable culture media for the growth of the typhoid bacilli, and from five of these tubes the germs were isolated. At the time that the collection was made from one of these privy vaults no fresh typhoid discharges were being emptied into it, so that the presence of the typhoid bacilli on the legs of the flies found there apparently proves that the germs already in the vault were alive and multiplying, or that the urine of feces of convalescing or recovered cases of typhoid still contained the typhoid bacilli.

We all know how flies swarm around privy vaults, cesspools, or other places where there is human excrement, and they have frequently been seen to fly directly from such places into kitchens and light upon food which was being prepared for eating. With undisinfected typhoid discharges being deposited in such places it can readily be seen how flies can infect food.

During last summer, in the town of Union Springs, which is supplied with water from an artesian well nine hundred feet deep, there occurred a case of typhoid the origin of which we were not able to satisfactorily explain. During the next two months there developed three cases in two families on adjoining lots. It so happened that none of the three persons who contracted typhoid saw the first patient referred to. They were drinking the same water as drunk by over three thousand other people, yet there was only one other case of typhoid in the town at the time, and they all had different milk supplies. The flies in that particular neighborhood were very numerous because of the fact that there is a livery stable on the next block, so that it seems probable that flies must have been the carriers of the infection in these cases.

The Army Board above mentioned makes the following statement regarding the dissemination of typhoid by flies among the soldiers in the Spanish-American war:

"Flies were undoubtedly the most active agents in the spread of typhoid fever. Flies alternately visited and fed on the infected fecal matter and the food in the mess tents. More than once it happened that when lime was scattered in the fecal matter in the pits, flies with their feet covered with lime were seen walking over the food. Typhoid fever was much less frequent among messes who had their tents screened than it was among those who took no such precautions. Typhoid fever gradually died out in the fall of 1898 in the camps as Knoxville and Meade, with the disappearance of the fly, and this occurred at a time of the year when in civil practice typhoid fever is generally on the increase.

"The first pits at Knoxville contained, before the first twenty-four hours had passed after the arrival of the troops, fecal matter infected with the typhoid bacillus. Flies swarmed everywhere. Instead of abating, the disease increased. The soldiers were using the same water used exclusively by the citizens of West Knoxville, and among the latter there was not at that time a case of typhoid fever."

*Dust.* It is perfectly possible for the typhoid bacillus to dry and become adherent to particles of dust and thus get into the atmosphere. Numerous observers have demonstrated that the typhoid germ can retain its vitality in dry dust for a variable length of time. Uffleman has shown that it may live as long as sixty days in dry soil, and in moist soil it may live and propagate for years. The soil may be polluted with the typhoid germ either by the urine or feces of a typhoid patient. It has been found that the feces of a person developing typhoid may contain the germs in large numbers before any symptoms arise to indicate that he has the disease. In from 20 to 30 per cent. of cases of typhoid fever the urine contains the germs. Gwyn reports ten cases of typhoid bacilluria in which pure cultures of the micro-organisms were found in numbers varying from 10,000,000 to 500,000,000 to the c.c. The urine is sometimes so full of the germs that it becomes cloudy. I have recently had such a case, and I may add that it resulted in death. In some cases the urine may contain the germ for months after the patient is well of the disease. So the typhoid fever patient before he is sick, and after he is well, may scatter the infection.

It is not probable that dust is carried for great distances in the atmosphere except in fierce winds, though dust is certainly one of the means of conveying the infection. Infected dust in rooms where typhoid fever patients are confined may cling to the hands, faces, or clothing of those exposed, or it may get into the secretions of the mouth and nose, and later be swallowed with food or water. When the floor is swept off or the rugs and carpets dusted, as is frequently done by people who are ignorant of the danger from infected dust in the typhoid sick-room, the germs may fall on food or milk, or even into the family's drinking-water, and those who have not been in the room may thus become infected.

Recently there have been several cases in which the typhoid germs have been inhaled into the lungs, there producing a true typhoid pneumonia, without any involvement whatever of the intestinal tract. One of these cases occurred at the Johns Hopkins Hospital, and was diagnosed and treated as an ordinary case of broncho-pneumonia. The autopsy showed the characteristic lesions of a broncho-pneumonia, with large numbers of the typhoid bacilli in the lungs, blood and spleen, but there was no lesion of the intestinal tract. There have been many other cases of typhoid fever reported in which there was no involvement of the intestines, though the Eberth bacillus was found in the lungs, blood, spleen, gall-bladder and meninges. In fact, there is much evidence to warrant the belief that the infection of typhoid is not infrequently taken into the lungs, and therefore we should guard against inhaling dust that has had the opportunity of becoming infected with the typhoid germ.

Eugene Wasden, of the Marine Hospital Service, asserts his belief that in the majority of cases of typhoid the lungs are the infectionatria, and that colonies of the bacilli first locate at some point in the respiratory tract, generally the lower lobes of the lungs. He says that in practically all cases typhoid sepsis occurs early in the disease, and that the lesions in the intestines do not occur until after the germs get into the blood. He reports his last series of (27) cases, in all of which he was able to demonstrate the primary pneumonic focus, and in them all he found the lung symptoms characteristic of a broncho-pneumonia or a subacute bronchitis. He thinks that dust is the great disseminator of typhoid fever, and remarks that "it would be better to filter the street-sprinkling water supply and allow the drinking of hydrant water."

As to the part that dust played in spreading typhoid fever in the concentration camps during the Spanish-American war in 1898, I will quote the following from the Army Commission's report:

"The shell roads through the encampment at Jacksonville were ground into the finest dust by the heavy army wagons. The scavenger carts carrying the tubs filled with fecal matter passed along these roads, and their course could often be traced by the bits of feces falling from the tubs. Other vehicles ground up the fecal matter and dust together and the winds disseminated these particles here and there. Men inhaled the dust. It was deposited in food in mess tents by the roadside, and men ate the dust. Pollution of the soil by those suffering with typhoid fever was of frequent occurrence. Cases of this disease under the diagnosis of malarial fever were repeatedly treated throughout their sickness by the regimental surgeons. Patients convalescing were also returned to their respective companies. Under these conditions there must have been abundant opportunity for contamination of the camp site with the specific germs. We are, therefore, inclined to the opinion that infected dust was one of the factors in the dissemination of typhoid fever."

*Contact Infection.* A number of physicians hold to the theory that typhoid fever is contagious in some unknown manner, and in upholding their position they point to the fact that it is a frequent occurrence for those who are nursing typhoid to contract the disease; and that with a water supply of undoubted purity, other persons in the house with a case of typhoid fever contract the disease.

With our present knowledge of the habits and life history of the typhoid bacillus, the origin of these can be easily explained.

It has been proven that typhoid bacilli can live and retain their virulence for two or three months on cotton, woolen, and linen cloth. Persons exposed to a case of typhoid may carry the germs on their clothing or person for weeks and thus contract the disease and carry it to others. Nurses, in handling the discharges and soiled linen or bedding of a patient sick with typhoid fever, may get the germs on their hands, and unless they are careful about disinfecting them, in handling their own or other people's food may also be the means of conveying the infection. The typhoid bacilli may live for weeks under the finger-nails.

Under the heading of dust I necessarily had to discuss some phases of contact infection and I will not dwell further on it, though in supporting the views which some may consider unorthodox, I can not refrain from quoting the following paragraph from the Army Board's report:

"It is more than likely that men transported infected material on their persons and in their clothing and thus disseminated the disease. We have condemned the method as followed in many of the camps of detailing men from the ranks to act as orderlies in the hospitals. In some of the commands it was customary to detail a hundred or more men from the line every morning. These men went to the hospitals and handled the bed-pans used by the persons sick with typhoid fever, and at night returned to their comrades. The most of these men were wholly ignorant of the nature of the infection or the methods of disinfection. In fact, at one of the division hospitals we saw orderlies go from the hospital and partake of the midday meal without even washing their hands. These men handled not only the articles of food which they ate, but passed articles to their neighbors. It seems to us that a more certain method of disseminating an infectious disease could hardly have been invented. Certain tents were badly infected and the majority of their inmates had the disease, while other tents wholly escaped. Blankets and tentage became soiled with the typhoid discharges, and in this way the disease was propagated and carried by the company wherever it went. We therefore believe that personal contact was an important factor in the spread of the disease."

*Uncooked Vegetables.* Celery, water cress, lettuce, and other vegetables which are eaten without cooking may become infected with the typhoid germ, either from the soil in which it is grown or from being washed in polluted water. Several epidemics from this source have been reported, notably the one at Northampton, Mass., in an insane asylum in which there occurred a large number of cases of typhoid where the source of infection was seemingly traced to eating celery which had been grown in beds fertilized with "night soil."

Oysters may become infected in their beds, or in polluted fresh water in which they are sometimes placed to undergo the process known as "plumping." A number of typhoid epidemics have occurred which were undoubtedly due to infected oysters. Last December at Portsmouth, England, fourteen of the prominent guests who had attended the mayoralty banquet developed typhoid fever. Upon investigation it was found that the Emsworth oyster beds, from which were taken the oysters served at the banquet, were located where the town's sewage was discharged directly over them; and as this sewage was almost certainly polluted with the typhoid germs, it seems certainly almost probable that the oysters became infected in that way.

*Ice.* Freezing does not actually destroy the typhoid bacillus, though it unquestionably inhibits its growth and virulence. However, it has been known to live and retain its virulence in ice for more than a hundred days. Manufactured ice is not likely to become infected, since it is made largely from sterilized water, but the ice from lakes, ponds, and streams, which is stored up in winter and kept until spring, is almost as likely to be infected as drinking-water from the same sources, though, as I have said, the cold of ice greatly lessens the virulence of the typhoid germs and in four months will destroy them.

The prevention of disease is the highest and noblest art in our profession, and when physicians and the laity realize that typhoid fever is a highly infectious disease and so become imbued with the importance of preventing its spread, then will we hope to make it one of the rare diseases. I fear that the majority of physicians are too negligent in the prophylactic management of typhoid fever. It would be better for the world if physicians sent for in cases of typhoid fever were to prescribe methods for preventing other members of the family from contracting the disease, and let the patient go without medicine, than to do as is frequently done by physicians who ought to know better, that is, dose the patient with medicines, which in the majority of cases do no good, and make no effort to find the source of the infection, nor use any methods to prevent its spread. That is a very sensible law in North Carolina, which makes it a criminal offense, punishable by fine or imprisonment, for a physician who is treating a case of typhoid fever to fail to instruct the attendants regarding the methods of disinfecting the discharges of the patient. If a physician has not the moral courage to do his duty in preventing disease there should be laws "to hand the wretch to order."

How can we guard against the infection of typhoid fever in sources other than drinking-water? I shall take the position that the place to begin is with the patient, because there you have a known focus of infection.

The typhoid fever patient should be isolated, as we do other infectious diseases, in a well-ventilated room, preferably on the sunny side of the house. Fresh air and sunlight are more potent disinfectants than many of those sold on the markets. Windows, curtains, pictures, and other hangings, carpets, rugs, and unnecessary furniture, which catch dust and hold the infection, should be removed from the room. His personal and bed linen, upon becoming soiled, should be immersed in an antiseptic solution before being taken from the room, and before use again should be boiled for not less than half an hour. The excreta should be carefully disinfected. Simple immersion in an antiseptic solution is not sufficient to destroy the typhoid germs in feces—it should be kept in contact with the solution for several hours. The bed-pans and urinals should constantly contain a small amount of the antiseptic solution, and the discharges should immediately be placed in another vessel containing at least two parts of the solution to one of the feces. The feces should be thoroughly broken up and mixed with the disinfectant solution, and should so remain for at least four hours before being placed in the water-closet, if the house is connected with a sewer; if not, it may be covered with dry earth at a place where the drainage will not go into the water supply. Under no circumstances should the excreta of a typhoid patient be placed in a privy vault, or exposed in any place where it can be reached by flies, or becoming dry get into the atmosphere as dust. In other words, do not depend entirely upon chemical disinfectants to destroy all the typhoid bacilli in the discharges of any patient suffering with that disease.

Urotropin given internally, in doses from five to ten grains every four hours, will completely sterilize the urine, and since in some cases of typhoid bacilluria the germs may be found in the urine for weeks after convalescence, the urotropin should in those cases be kept up for several weeks after the patient's recovery. Probably the best chemical agent for disinfecting urine after it has been voided is formalin (a 40 per cent. solution of formaldehyde in water). One teaspoonful added to half a pint of urine will completely sterilize it in a few minutes. For disinfecting feces with formalin use two parts of a solution containing one tablespoonful of formalin to one quart of water to one part of feces.

Carbolic acid (3 per cent. solution), preferably with from 3 to 5 per cent. of common salt, or with a small quantity of a mineral acid, next to formalin is probably the best disinfectant for destroying the germs in typhoid excreta, and has the advantage of being cheaper than the formalin.

The cresols, which occur as impurities or by-products in the manufacture of phenol and more powerful germicides, and less toxic to man than the carbolic acid, and since they are also cheap, are coming into general use. Creolin, which contains 10 per cent. cresols held in solution with soap, is a very effective germicide when used in a 5 per cent. solution. Lysol contains 50 per cent. of cresols with neutral potash soap, and in a 1 per cent. solution is as powerful as a 5 per cent. solution of carbolic acid. The crude carbolic acid, since it contains as an impurity the cresols, is preferred by some, who claim that it is not only cheaper but a more efficient germicide than either the pure carbolic acid or the cresol preparations.

Mercuric chloride is used perhaps more than any other agent for disinfecting, and is one of the most powerful antiseptics that we have, yet in disinfecting feces it is probably almost inert. It forms upon the addition of organic matter the albuminate of mercury, which coats over the substances and temporarily inhibits germ life, but it has very little powers of penetration. With feces it likely forms the sulphide of mercury, which has very slight bactericidal properties, before it has time to have much effect in destroying the bacilli in the feces. The use of mercuric chloride in typhoid fever should be limited to disinfecting the hands and in washing off the floors, walls and furniture.

Nurses after handling the patients' soiled linen, or bed-pans and urinals, should use great care in disinfecting their hands, particularly before taking food. Simply washing the hands is not sufficient ; the space under the nails should be carefully cleansed, and the hands after thorough washing in soap and hot water should be bathed in an antiseptic solution, preferably a 1 to 1,000 solution of mercuric chloride. Those who are nursing a typhoid patient, or who are constantly in the room with him, should change their clothing before coming in contact with others.

After the death or recovery of a patient the room should be disinfected as in other infectious diseases.

In guarding against milk infection it is well worth the while for every family to examine carefully into the milk supply. Dairies which supply milk to cities should be regularly inspected by an efficient medical officer. Cows should not be pastured on lowlands upon which the sewage of small towns is drained. Milk vessels should always be washed in boiling water before being used. Milkmen should use great care in cleansing their hands and the udders of cows before milking. In case of an epidemic of typhoid fever, or even with a single case in a family, it is best to boil the milk and protect it from the dust and flies after it is thus sterilized.

The proper disposal of the excreta of the typhoid patient will in a great measure prevent infection from dust. However, it should be remembered that the sputum of the typhoid patient may contain the Eberth bacillus, and it should be burned or disinfected as we do in tuberculosis of the lungs.

Flies present a greater problem. Of course, if the excreta is disinfected and placed where the flies can not reach it, they can not carry the infection; but every fly should be regarded with suspicion, because our neighbors, even at considerable distances from our homes, may not take any precautions in disinfecting the excreta of their typhoid sick. We should therefore screen with great care our windows and doors, particularly in the rooms where food is kept, prepared, or served. Something may done to destroy flies in their breeding places, which is largely in the excreta from horses, though they breed to some extent in human excrement. Cities should adopt ordinances requiring the proprietors of livery stables, and individual owners of horses, to screen the piles of horse manure, or to keep them covered in such a way that flies can not breed in them. Chloride of lime, which will destroy the larvæ of the house fly, should be kept in stables by the barrelful, and every few days should be sprinkled over the horse manure. These methods of lessening the number of flies are practical and inexpensive, and should be carried out on farms as well as in cities.

Typhoid infection from uncooked vegetables would be very much lessened if the practice of using human excrement, or "night soil," as a fertilizing agent were not permitted, particularly in growing vegetables which are eaten without cooking. Before eating any raw vetables they should be thoroughly washed in water of known purity, and in boiling water where it will not destroy their flavor.

Oysters which are suspected of being infected should never be eaten raw. Oyster beds should not be allowed near the openings of city sewers, and oysters in being "plumped" should be placed in water of known purity.

"Ice, in its manufacture, should be made only of pure water, and that which is obtained from ponds, lakes, and streams should not be cut near the outlets of sewers, or at other places where the water is likely to be polluted. However, ice is such an infrequent source of infection for typhoid fever that we may almost wholly disregard it as a factor in the production of the disease.

Finally, as I have said before, the prevention of typhoid fever should begin by limiting the infection to the patient under treatment; and when physicians do their full duty in instructing the laity regarding the infectiousness of typhoid fever, as well as to see that proper methods are carried out to prevent its spread, we may almost hope that typhoid fever will cease to be one of our endemic diseases.

Union Springs, Ala.

---

### THE INDIVIDUAL EQUATION OF THE PATIENT IN SURGICAL OPERATIONS.*

Aseptic methods have so minimized the immediate danger of surgical procedures, and modern surgical technique has given the surgeon such facility in operating, that, nowadays, operations are undertaken lightly and performed with comparative safety, and with very little anxiety as to the recovery of the patient from the operation itself, in the majority of cases. Occasionally it happens, however, that a patient dies upon whom an operation has been done of a kind which the surgeon has performed for others many times without any serious result, and what seems a perfect carrying out of a thoroughly well-planned and an ideal operation; also without any manifest contraindication on the part of the patient.

I am sure every surgeon who does much operative work has had occasion to ask himself in a given case, why did that patient die when so many other patients have recovered who seemed in no better condition and who, in many instances, were actually less able to stand the operation than he was? I believe the answer to this question is, "It was on account of the individual equation of the patient." The fact that there is an average result amongst a large number of individuals operated on naturally suggests that there is an average vital personality. There are many exceptions to this rule, however. I think surgeons ought carefully to consider these possible exceptions in undertaking any operation of expediency and bear them in mind in prognosing.

It is my purpose to call attention to a few conditions which may

---

* Read by William L. Estes, M.D., of South Bethlehem, at the meeting of the Medical Society of the State of Pennsylvania, held at New York, September 22-24, 1903.

affect the reaction of an individual to a traumatism and which may vitally affect the result of an operation.

The first of these conditions is heredity. Barring the exanthemata, in which class I may for the instance place syphilis, it is probable that no disease is actually transmitted as such from parent to child. It has been abundantly proved that individual proclivities both of physique and disposition are transmitted to children from parents (there are probably just as many instances of atavism in the matter of physical qualities as there are in physical appearances and mental qualities of an individual). There is, further, much evidence to show that there is a chemical individuality which may be transmitted as a family trait. The well-known instances of hemophilia belonging to certain families for a number of generations would indicate this. It is of great importance, then, for the surgeon to find out the family history of his patient.

The manifestations of heredity in the second generation of syphilitic, of tubercular and of cancerous families are so nearly related that it seems in some instances they are interchangeable. That is to say an individual whose parents have had syphilis seems as much disposed to cancer as one whose parents have had cancer. Such an inheritance also renders the child as liable to tuberculosis as if he inherited the tendency directly. I am not able authoritatively to speak in the converse, that is, I have not sufficient evidence to show that the tubercular and cancerous inheritance also disposes towards syphilis. I believe, however, it would only be fair to extend the parity of reasoning thus far. A cancerous inheritance undoubtedly disposes towards tuberculosis, and a tubercular inheritance disposes towards cancer. This all means that the physiologo-chemical resistance of the cells and tissues are so reduced by an inheritance of a diathesis that the individual is particularly susceptible to microbic inoculations. (Incidentally this may be used as another argument for the microbic origin of cancer.)

All surgeons know that individuals react very differently to sepsis. Some are overcome at once, while others bear an exceedingly virulent invasion with comparative safety, and others still occupy a middle ground with reference to vicious micro-organisms. Some individuals after operations recover from most exaggerated conditions of septic peritonitis brought on by fulminating and gangrenous appendicitis or salpingitis, while others apparently equally strong in every way, succumb to a very mild form of sepsis even after the most careful and painstaking operation and unremitting care and skillful after-treatment. It is true that all apparently mild conditions of sepsis from appendicitis are really far from being such, as they may possess the potential features of a most virulent and fatal inoculation. On account of a pyelo-phlebitis of the distal branches of the portal vein, thrombosis of these branches may, and frequently does, exist in cases of abdominal sepsis. Subsequent detachment of the emboli laden with strepto and staphylococci, and their lodgement in the liver or in some nearer small vein, may serve to determine many of the so-called "inexplicable deaths" which follow "ideal appendectomy." (Dr. A. G. Gerster, in a paper before the last meeting of the American Surgical Association, very forcibly demonstrated this fact.) But why do some cases escape these several manifestations of advanced sepsis, while others, with, as nearly as human foresight and experience procure, the same careful technique and management, die on account of this blood-poisoning? I think but one answer may be given to this, namely, individuals inherit or acquire varying powers of resistance. Some persons have undoubtedly inherited comparative immunity on account of the superior qualities of the antitoxins and cystolysins of their blood and tissues. That environment and habits also have to do with this is no doubt true, and I will mention this point under another head presently. As Darwin has shown, however, it requires many generations to change materially the outer form and characteristics of an animal by environment. We may fairly conclude that the quality of the cells do improve with the general improvement of the physique of the individual, but there is abundant evidence to show that the apparently most robust individual is not always the most resistant to septic conditions. If there were time I might cite numerous instances of this fact, but I am sure it is thoroughly familiar to you all. To sum up this head in a postulate I would say dyscrasias may be transmitted by inheritance and weaken the resistance of animal cells to toxins; individuals who have such an inheritance are particularly liable to microbic inoculations, and do not develop antitoxins and cystolysins of sufficient potency to overcome infections. Such individuals are apt to die from very mild forms of sepsis after operations.

Another circumstance which materially affects the result of operations is:

*2d. The Environment and Habits of the Patient.* It is not necessary to argue this point from any profound scientific basis. It is now commonly conceded that human beings in common with all animals, are markedly modified by their continued surroundings and their modes of living. In the ultimate scientific conclusions this belongs properly to heredity. I wish rather to call attention to some ordinary observations which bear on this matter.

It will be conceded at once that individuals who have habitually and for long periods been subject to very bad sanitary and unhygienic conditions do not bear operations well. My observations and experience have convinced me, however, that the bad conditions of this kind are not confined to city and town life. The advantage of free outdoor life with pure air during the daytime in rural life, is in many instances more than overbalanced by the lack of ventilation and crowding in the sleeping apartments at night, and the very common habit of eating poor and improperly cooked food, which is prevalent amongst farmers. Farmers eat a great deal of pork, salt, and dried meats and canned vegetables. They have the advantage, it is true, of fresh fruits in their seasons, but they use these fruits without discretion, usually immoderately while they last, and they very rarely ever have their meals properly cooked. There is no class of people, in my experience, so given to indigestion and the varying concomitant intestinal disturbances as farmers are. The children are commonly poorly nourished and only in early adult life do they seem near the proper standard of development and nourishment. Farmers are so poorly nourished and their digestive organs are usually in such wretched condition that they are not good subjects for severe operations and they are very liable to septic inoculation.

City people, besides being better fed as to quality of food, have their meals better prepared, as a rule, except in the very poorest classes, and they have, on account of daily necessity, acquired a certain degree of immunity to sepsis. Their antitoxins and cystolysins are continually stimulated and are in constant use. They are not so liable to septic developments as farmers are therefore. Except the very poorest people, who live in the congested tenement districts, who are subjected to foul air and surroundings, and who live on scanty and poor food year in and year out, I think city and town-reared people stand operations better than the ordinary farmers do.

Besides the environment, *the habits of the individual* have much to do in determining the result of an operation. The excessive indulgence of any appetite lowers the resistance of the whole system to trauma. I have found especially that excessive indulgence in beer markedly increases the tendency to phlegmonous inflammations. Beer drinkers furnish most fertile soils for streptococcal development. They are also most unfavorable subjects for general anesthesia.

Whisky drinkers are not only difficult to anesthetize but they are very subject to post-operative nephritis and pneumonia and delirium tremens, and on account of frequent atheroma they bleed more and they are more apt to die from so-called exhaustion after operation.

Vegetarians, or persons who live chiefly on vegetables, do not stand the loss of blood well, and they are peculiarly liable to die from acute anemias. They also react very poorly to saline infusions for the restoration, or the substitution, of the circulation of the blood in cases of exhaustion after severe acute hemorrhage.

On the other hand, meat eaters, the full-blooded and plethoric individuals, do not as a rule, stand general anesthetics well. It requires much skill to bring them under the anesthetic and constant and very skillful care to prevent asphyxiation when fully under, especially when ether is used. If the heart be absolutely sound chloroform should be the anesthetic of election in these cases. Perhaps Dr. Willy Meyer's "Anæsthetol," a combination of ether, chloroform and alcohol, may prove a boon in this class of cases, if further and extended use proves it to be really a genuine chemical combination, a new chemical substance, and not a mere mixture of anesthetics, subject to variations on account of the varying volatility of the combined drugs.

*3d. Disposition of the Patient.* Unquestionably, I think, a bright, cheery, sanguine, and courageous disposition tends markedly to favorably influence the result of any major operation. I always feel handicapped in operating on a despondent and excessively anxious subject. Nervous energy counts tremendously in the reaction, resistance and tenacity of an individual after an operation. This has more to do with the outcome of a severe operation than mere physical fitness. I hesitate long and undertake with many misgivings a serious operation on a person who "knows he will die from the operation." Such individuals do recover many times undoubtedly, but if aught goes seriously wrong their mental laxity and lack of nervous tone almost surely bring them to the grave.

*4th. Age.* The extremes of age increase markedly the bad prognosis of an operation. Very young children and very old people do not bear anesthetics well, nor can they stand the loss of blood. Such individuals are apt to die after long operations, and operations done for very vascular tumors or in regions where complete hemostasis is impracticable. Of 85 deaths after operations which have occurred in St. Luke's Hospital the last ten years, 7 were under 5 years of age; 10 were 5 to 20; 23 were 20 to 40; 30 were 40 to 60, and 15 were over 60 years of age. These I presume are average statistics, and while the number is too small to make any valuable generalization, they indicate that, barring the extremes, the exact period of life itself when an operation is endured does not make any material difference in the result.

*5th. Sex.* This seems to have no marked influence in the result of an operation provided the physical condition is good.* I think my experience has shown, however, that women bear operations for septic conditions of the peritoneal cavity better than men do. Probably the superior vascularity of the pelvic region in women, which is most frequently the seat of septic inflammations and accumulations, will account for this. Another fact may also influence

* Taking out 15 deaths after operation done for bad crushes, which were practically all men, my statistics show that 37 males and 33 females died out of 85 fatal cases.

this result, namely, that men use the diaphragm much more than women do in breathing. The action of the diaphragm tends to draw, by intermittent pressure and relaxation, the lymph current upwards. In men, therefore, septic products are much more apt to ascend to the subphrenic and supraphrenic lymph nodes, a lodgment which is well known to be especially apt to cause fatal results.

*6th. Nationality of the Patient.* This in my experience makes little difference in the result of operations. I have observed, however, that the lower classes of Slavs and Italians, such as are met with in this part of the country, are apt to be very emotional, and are very intolerant of pain, and are very difficult to restrain after serious operations. Crying and extreme restlessness must, of course, make great difference after some operations, and on this account these two classes of patients are not desirable subjects for major operations.

*7th. Physical Condition of the Patient.* The most important point for the surgeon to find out, and accurately to gauge, is *the physical condition of the patient at the time of the operation.* So-called "Laboratory Methods" have greatly broadened the scope of the former routine of physical examinations. The accuracy of the determinations of the character of execretions of the blood, and of most of the secretions, nowadays, in the laboratories, gives us certain data which are of immense value. I believe, however, surgeons are inclined to depend too much upon laboratory investigations and to neglect the old methods of bedside examinations, or at least, they relegate them to secondary importance. I believe that in no other field of medicine do the methods of Zadic so constantly apply, and are of such constant value as in surgery. In some instances, indeed, the findings of a personal physical examination should supersede or qualify the determination of a laboratory conclusion. For instance laboratory investigations tell us that an operation performed on a patient whose hemoglobin is below 40 per cent. is usually fatal. Yet I am sure we have all had uninterrupted recoveries and no serious result after many operations done on patients whose hemoglobin was below 40 per cent. It is true that individuals who have some cachexia and on account of this slow poisoning and undermining of their tissues and gradual deterioration of their blood, and who have less than 50 per cent. hemoglobin, are not safe subjects for operations. It is a different matter, however, if this lowering of the hemoglobin comes from a hemorrhage or from a series of hemorrhages.

Again it is conceded that albumen and granular casts in the urine indicate that the patient who excretes such urine is a very unsafe subject for anesthesia, especially ether anesthesia, and markedly increases the danger of *any* operative procedure. Large ovarian and uterine tumors are apt to produce, on account of pressure and mechanical interference with the blood supply, and the circulation in the pancreas, liver and kidney (chiefly the portal system), just such indications in the urine; the affected organs are relieved and the vitiated renal excretion is entirely remedied by the removal of the tumor, and the operation does not prove extra hazardous in these cases. I might multiply instances of this kind.

I wish to urge the great importance of a careful and thorough physical examination of every patient *by the surgeon himself*, or by some one qualified to make careful physical examinations. This should be done before even minor operations of expediency. Subjective symptoms may sometimes clearly indicate the seat of a lesion requiring surgical interference, in other cases it requires a careful systematic physical examination to find the disease. For instance, rectal carcinoma is frequently attended by very decided gastric symptoms. I have observed this in a number of cases. In one case, notably, the gastric symptoms were so pronounced that for months the patient was treated by some of the best internists for gastric disease. After the carcinoma was found and removed the stomach symptoms disappeared entirely.

It is a matter of serious importance that scarcely one medical school in the United States teaches its students to use their *eyes* in examining a patient. Students are laboriously indoctrinated into the importance of carefully cultivating their senses of touch and hearing, and are taught thus how to discriminate between normal and abnormal protuberances and sounds of the body, but we scarcely can find a student who thinks of first carefully inspecting the surface of his patient's body or his extremities in order to *see* and thus note any abnormalities, and to know how to discriminate between the normal and abnormal contour and color, or tension of the body. Many instances have come under my observation where this lack of training has led to serious errors in diagnosis. Unquestionably, I think, both personal, clinical and laboratory examinations and investigations should be made, whenever it is possible to have them done, before all serious operations.

Organic diseases of any of the viscera greatly modify the prognosis of operations. Dr. J. R. Bradford, in the *Lancet*, April 4th, 1903, calls attention to certain so-called "latent diseases." That is to say these diseases are of such a kind that they might exist in a given case without any special evidence of their presence, and unless one makes a careful, painstaking examination they might not be recognized. Operations done for some other cause might be particularly hazardous on account of those latent diseases. The diseases mentioned by Dr. Bradford are: 1st, nephritis; 2d, diabetes mellitus; 3d, typhoid fever; 4th, rheumatism; 5th, latent cerebral abscess, from middle ear disease; 6th, pleural effusions; 7th, gastric ulcer; 8th, malignant disease of the stomach; 9th, gallstones; 10th, the rare conditions of a high degree of atrophic cirrhosis of the liver in children. This condition may produce symptoms of acute peritonitis. All these diseases may be excited to active conditions from the latent state by any operative procedure and may prove disastrous to the patient.

General arterial atheroma, or entarteritis deformans, also contributes markedly to the danger of an operation. When this condition is accompanied by an intermittent heart action, no matter how well the patient may otherwise seem to be, the prognosis after any serious operation is very grave; the coronary arteries are involved and the heart is fatty.

As was suggested before, patients who are already in serious septic states, especially those who have septic peritonitis, are very bad subjects for operations. The reason is in these septic conditions it is frequently impossible to get rid of all the septic matter. There is frequently pylephlebitis of the branches of the portal vein or infection of the posterior lymph nodes of the peritoneum. The most thorough washing out and drainage will not relieve the patient in these cases. However, the all important point *is thorough drain-*

*age, after* thorough removal of the septic products, and thorough cleansing. The posterior or lumbar route should be used for men, the vaginal for women. Fowler's position is good for women especially, for men lumbar drainage is far better. Of the 85 deaths after operation which I have had in the last ten years, 40 of the patients were already septic.

Patients who are seriously affected by malaria are also bad subjects for operation, so are also leucocythemic patients.

It is the duty of the surgeon to find out the existence of *any* organic disease, and, reasoning from what he knows of its nature and tendency, he should apply this in estimating the effect in his proposed operation.

*8th. The Extent and Locale of the Operation.* Aseptic surgery, and the technique of thorough hemostasis have robbed extensive operations of much of their danger. There are no "*Nole me tangere*" regions in the human body now. Still there are two regions which seem to be especially unfavorable for extensive operations. I refer to, 1st, the base of the brain, and, 2d, the throracic cavity. With great care even these two regions may be invaded without serious result, however. For instance, I have recently had two very extensive crushes of the cranium which involved the base on both sides as well as the vault, and which required exploration of the base of the brain on both sides, make rapid recoveries even though there was great hemorrhage and injury to the cerebrum. I have also removed a large segment of the whole thickness of the thoracic cavity in an operation for a secondary scirrhus, and sewed the lung to the opening in the walls of the thorax, and finally, by using the external surface of the lung to block up this opening, have succeeded not only in saving the life of a cachetic woman but also secured an excellent functional result for both lung and thorax. Notwithstanding these and many other good results (notably the closing of many wounds of the heart), which other surgeons have had in these regions, the fact remains that these two are perhaps the least favorable of all the regions of the body to invade. So one must always bear this fact in mind in estimating the chances of any case which requires operations in these regions. The scope of this paper will not permit me to go into the reasons for the bad prognosis of operations on the base of the brain and in the thoracic cavity. I must refer my hearers to modern text books for further discussion of the matter.

Thus I have tried to show that,

1st. Inheritance.

2d. Environment and habits.

3d. Disposition of the patient.

4th. Age of the patient.

5th. Sex of the patient.

6th. Nativity of the patient.

7th. Physical condition of the patient at the time of operation.

8th. The extent and locale of the operation.

All have something to do in determining the personal equation of the patient about to be operated on. I insist again it is the surgeon's duty conscientiously to endeavor to *find all the data* which will enable him to determine the importance of all these points with reference to any individual upon whom he intends to operate. This means he must take a careful history of the patients including his family history, as far as it may be definitely obtained. Also he must make a careful and thorough physical examination, and call to his aid laboratory investigations. With an experienced and careful surgeon I am disposed to lay the greater importance on the *personal physical examination*, and should rather trust to this than to laboratory findings, to determine the possible danger of any proposed operation, taking for granted, however, that an examination of the urine shall be one of the examination, included in my term personal physical examination.

Reflection on this subject and these facts which so critically influence our operations suggest that more difficult than determining *when to operate* is to know *when not to operate*, and this last is to the patient, in many instances, far more important. Our age is inoculated and thoroughly intoxicated with the furor for operations. I think it is time for thoughtful, educated surgeons to study more the *prevention* of operations than new operative procedures.

Preventive surgery is just as possible and important as preventive medicine. Let us try, for instance, to learn the *cause* of the various surgical conditions and then try to remove these causes and so prevent the disease. As Sir W. H. Rennett has properly said, the majority of surgical operations come from the three most prevalent diseases: 1st, cancer; 2d, tuberculosis; 3d, syphilis. If surgeons would try faithfully to prevent these diseases many fewer operations would be necessary.

We do not yet know the actual agent which causes cancer. We do know, however, that constant irritation of a part, especially where mucous and cutaneous surfaces are in juxtaposition, will produce epithelial cancers. Forbidding the use of a short stem pipe, or any smoking, the filing of the sharp edge of a tooth may prevent epithelioma of the mouth and lips. Attention to little ulcers of the anus, the early amputation of a degenerated uterine cervix, which has been previously neglected after it has been lacerated, may prevent cancers of the rectum and uterus.

Further, it is generally conceded by pathologists, as well as surgeons, that even after a lesion has started there is a period which may be called the precancerous period; during this period a tumor which may subsequently be most malignant has the qualities of a benign tumor, and it may be removed with assurance that it will not recur.

It is of the greatest importance that every tumor which requires an operation shall be removed as soon as it develops. People generally are not thoroughly impressed with the tremendous importance of this fact. Surgeons should endeavor to educate the people to a thorough appreciation of this. By educating the people, tuberculosis is finally regarded as a disease which may be prevented, and people have been taught and are being taught what measures to take to prevent this dread disease. Its half brother, and the far more dreadful, cancer, may also be prevented, at least in some instances. Surgeons first should teach family physicians, and they in turn their families, how to prevent and stop the progress of cancer.

Temporizing in surgery will not do. People should be taught that when they have any tumor or lesion of any kind which requires removal or operative interference, it is of vital importance to have it done at once. Physicans frequently do not believe this themselves, else surgeons would have far less trouble with cases which have "gone too far."

In conclusion, I beg to appropriate the words of Sir W. H. Bennett from the *Lancet*, May 23d, 1903: "In the meantime, taking things as they are, it is well that we should beware lest a single predominant factor should be allowed to lead to our regarding, through a small tube only, a subject the horizon of which is absolutely unlimited. It has been said that the basis of surgery is handicraft, and this, in a sense, is true; but surely it is truth only half told, for apart from the issues to which I have referred there is lying behind a far greater thing, the knowledge of when to apply that craftmanship of which every one who now aspires to the practice of surgery should make himself a master. Nothing that has happened in the improvements connected with the practice of our art justifies, as far as I know, the modification by one iota of the edict of the great surgeon, who before advancing science had robbed operations of most of their horror said, 'The all important thing is not the skill with which you use the knife, but the judgment with which you discern whether its employment is necessary or not.'"—*Penn. Med. Journal.*

---

SOME IMPROVEMENTS IN THE METHOD OF LOCAL ANALGESIA.

A clinical lecture with the above title, delivered at University College Hospital, London, July 11, 1903, by Arthur E. J. Barker, F. R. C. S., Eng., appears in *The Lancet* for July 25, 1903.

Several points must be borne in mind, among them the mechanical and physical difficulties in infiltrating all the nerves supplying an extensive field of operation. To inject the whole area so as to reach all its nerves would mean in many cases the use of much more of the toxic drug than is necessary, and in some cases so much as to be dangerous.

The author refers to certain observations by Braun, of Leipsic, on a method of overcoming the drawbacks incident to the usual mode of producing local anesthesia. This method is based upon the old experience that *anything which retards or diminishes the circulation of the blood in a part enchances the potency of the analgesic agent.* Experiments were made with Adrenalin, a very small quantity of which was injected with B–eucaine (or cocaine) into the author's own arm, and subsequently into the arms of numerous patients. After the lapse of twenty minutes the part was quite blanched and wholly insensitive to pain, remaining so for about two hours. Adrenalin alone, used in this way, had no analgesic effect, while the results of the use of the combined solutions of B–eucaine and Adrenalin were far superior to those produced by B–eucaine alone.

The most convenient way to prepare the solution is as follows: Powders each containing O, 2 gramme (3 grains) of B–eucaine and O, 3 gramme (12 grains) of pure sodium chloride are kept in thick glazed paper, ready for use. When needed, one powder is dissolved in 100 Cc. ($3\frac{1}{2}$ fluid ounces) of boiling distilled water and 1 Cc. of Parke, Davis & Co.'s Solution Adrenalin Chloride is added when the fluid is cool. The solution is left in the Jena glass beaker in which it has been boiled, which is carefully covered and placed in a vessel of warm water to keep it at blood heat.

The injection is made by means of a simple syringe of glass and metal of 10 Cc. capacity, with rubber washers, which can be sterilized by boiling.

To illustrate his method the author describes in detail the performance of an operation for the radical cure of inguinal hernia. The hernia is first reduced and the index finger is thrust into the external ring as far as possible. Along this finger the needle is entered and the inguinal canal is filled with 10 Cc. of the solution. An endeavor is made to inject it all around the neck of the sac so as to reach the genital branch of the genitocrural nerve. The needle is then entered at the external end of the line of incision in the skin, and is made to infiltrate the *superficial* layers of the latter down to the root of the scrotum, making the resulting wheal at least an inch longer at each end than the incision is to be. Injections are then made at a point half an inch to the inner side of the anterior superior spine of the ilium, the needle being thrust towards the ilio-inguinal nerve, and at a point about one inch above the middle of Poupart's ligament where the ilio-hypogastric nerve is most conveniently met. Then the thigh is flexed and another syringeful is injected along the ramus of the pubis and the root of the scrotum or labium.

It is necessary to wait twenty minutes after the last injection for the full effect of the Adrenalin to develop. The whole field of operation should be blanched and insensitive to pricks, but not to touch—analgesia, not anesthesia. The incision may then be made with confidence that no pain will be felt. The absence of oozing of blood is noticed. Only large vessels bleed at all.

Success depends upon a mastery of the principles, and practice in the details of the method. It is not enough to inject the fluid under the skin generally. Due regard must be had to the position and course of the nerves supplying the structures to be dealt with. The Adrenalin compound, by slowing the circulation through the part prevents the anesthetic agent from being rapidly washed away. The writer has used this method in thirty operations including the radical cure of hernia, strangulated hernia, orchidectomy, removal of varicose veins, psoas abscess, loose body in knee, tumor of neck (actinomycocis), colotomy, thiersch skin grafting and cystic adenoma of the thyreoid.

